# Beyond STMN1: stathmin-family control of microtubule dynamics and paclitaxel resistance in cancer

**DOI:** 10.3389/fonc.2026.1940719

**Published:** 2026-09-01

**Authors:** Haoyu Li, Lizhou Shi, Qinghua Wang, Wei Han

**Affiliations:** 1 Department of Gastroenterology, Kunshan First People’s Hospital Affiliated to Jiangsu University, Kunshan, Jiangsu, China; 2 Department of General Surgery, Kunshan Women and Children’s Healthcare Hospital, Kunshan, Jiangsu, China; 3 Department of General Surgery, Kunshan First People’s Hospital Affiliated to Jiangsu University, Kunshan, Jiangsu, China

**Keywords:** chemoresistance, microtubule dynamics, paclitaxel, stathmin, STMN1, STMN3, taxane resistance

## Abstract

Paclitaxel is a cornerstone microtubule-stabilizing agent, but intrinsic and acquired resistance limit durable benefit. Resistance emerges from interacting changes in drug transport, tubulin composition, microtubule regulation, mitotic fate, apoptosis, autophagy, cellular plasticity and the tumor microenvironment. The stathmin family—STMN1, STMN2, STMN3 and STMN4—controls microtubule assembly through tubulin sequestration and catastrophe-promoting activity and therefore occupies a direct functional interface with paclitaxel pharmacodynamics. STMN1 has the strongest evidence: overexpression can reduce taxane sensitivity, whereas suppression restores microtubule stabilization and drug response in several experimental systems; clinical studies also associate high STMN1 with unfavorable outcome or reduced taxane benefit in selected cancers. Evidence for STMN3 is narrower but includes a mechanistically relevant ovarian-cancer study in which bisphosphorylated PEA-15 sensitized cells to paclitaxel by attenuating SCLIP/STMN3-mediated microtubule destabilization. By contrast, direct evidence for STMN2 and STMN4 in taxane response remains insufficient. This review develops a microtubule-centered resistance model, distinguishes established findings from context-dependent observations and testable hypotheses, and evaluates biomarker and therapeutic strategies targeting stathmin expression, phosphorylation, stability and protein interactions. We propose that stathmin activity is most likely to have clinical value as part of a composite taxane-response classifier that also incorporates intracellular drug exposure, tubulin isotypes and apoptotic competence.

## 1 Introduction

Paclitaxel is a foundational microtubule-targeting agent used in breast, ovarian, lung, gastric, pancreatic and other solid tumors. By binding to the taxane pocket of β-tubulin within assembled microtubules, paclitaxel suppresses normal microtubule dynamics, interferes with spindle function and chromosome segregation, and can drive prolonged mitotic arrest, mitotic catastrophe, senescence or apoptosis. The biological outcome is context dependent: some cells die during mitosis, whereas others undergo mitotic slippage and survive as genomically unstable progeny. This diversity helps explain why an identical exposure may produce durable tumor control in one setting but only transient growth inhibition in another (1–6).

**Figure 1:**
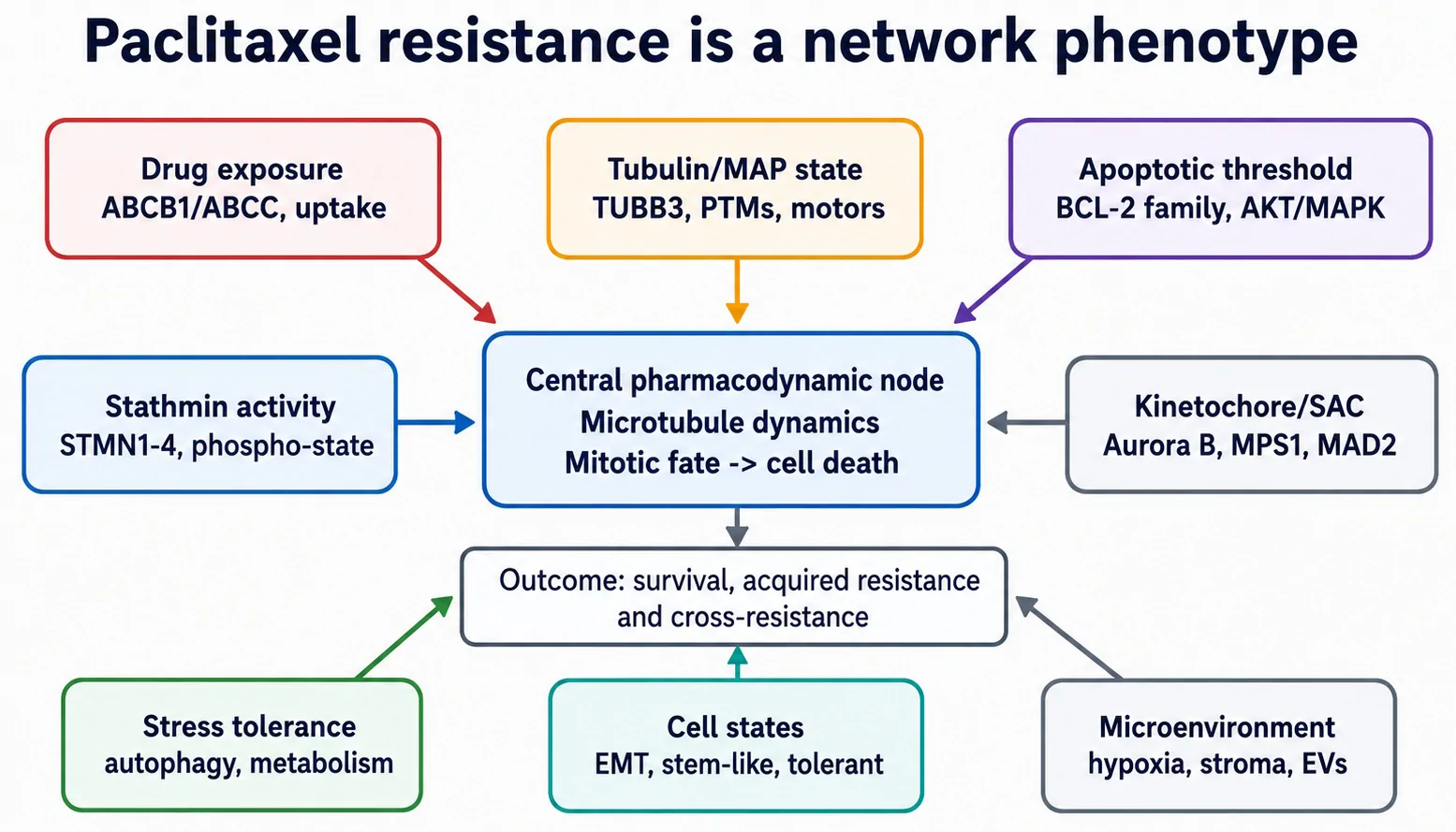
Input-to-outcome network of paclitaxel resistance. Paclitaxel resistance emerges from interacting layers that modify intracellular drug exposure, tubulin and microtubule-associated-protein composition, stathmin-family activity, kinetochore/spindle-checkpoint adaptation, apoptotic priming, protective stress responses, cellular plasticity and microenvironmental support. These inputs converge on paclitaxel pharmacodynamics, microtubule dynamics, mitotic fate and cell death, producing persistent disease, acquired resistance and cross-resistance.

Resistance can be intrinsic, evident before treatment, or acquired through selection and adaptation during repeated exposure. It rarely results from a single lesion. Instead, resistant tumors commonly combine reduced intracellular drug exposure, altered tubulin composition, rewired stress responses, defective apoptotic commitment, epithelial–mesenchymal plasticity, stem-like cell states and microenvironmental protection. A clinically useful model must therefore connect drug transport, the physical state of the microtubule cytoskeleton and cell-fate decisions rather than treating them as independent lists of mechanisms (2–6). The major input-to-outcome layers of paclitaxel resistance are summarized in **Figure 1**.

The stathmin family provides a biologically coherent point of integration. STMN1, STMN2, STMN3 and STMN4 share a stathmin-like domain that binds soluble α/β-tubulin heterodimers and regulates microtubule assembly. STMN1 is a broadly expressed cytosolic protein, whereas STMN2–4 contain N-terminal extensions that support membrane association and distinct subcellular localization. In their active, relatively dephosphorylated states, stathmin-family proteins can sequester tubulin and promote microtubule catastrophe. They do not compete with paclitaxel for the same binding site; rather, they functionally counter-regulate the microtubule-stabilizing state that paclitaxel seeks to impose (7–14).

Evidence for this relationship is strongest for STMN1. Experimental overexpression can decrease taxane sensitivity, whereas RNA interference or other forms of STMN1 suppression can restore microtubule stability and enhance responses to paclitaxel or related agents. Clinical studies in gastric, lung and endometrial cancers further suggest that high STMN1 protein levels may identify tumors with unfavorable outcome or reduced benefit from taxane-containing therapy, although study design, assay thresholds and treatment regimens vary substantially (15–24). More recent work has identified upstream stabilizers of STMN1, including PPP1R14B, thereby linking microtubule destabilization to drug resistance in triple-negative breast cancer (21).

The less studied family members require more careful interpretation. STMN3, also called SCLIP, has established microtubule-regulatory activity and has been connected to tumor-cell morphology, motility and several signaling networks. A direct mechanistic study in ovarian cancer demonstrated that bisphosphorylated PEA-15 can enhance paclitaxel sensitivity by impairing the microtubule-destabilizing effect of SCLIP, providing an important proof of principle that a non-STMN1 family member can influence taxane response (25). Nevertheless, evidence is sparse, tumor specific and often indirect. A 2026 review summarized the broader roles of STMN3 in cancer, including paclitaxel chemosensitivity (26). The present review therefore adopts a distinct, family-wide and drug-focused perspective: it critically evaluates how the balance between paclitaxel-induced microtubule stabilization and stathmin-mediated destabilization may shape resistance, distinguishes established observations from hypotheses, and identifies translational barriers that must be addressed before STMN proteins can be used as predictive biomarkers or therapeutic targets. This narrative synthesis was structured to provide balanced evidence appraisal and transparency consistent with SANRA principles (27).

## 2 Paclitaxel action and the architecture of resistance

A useful framework begins with the pharmacodynamic lesion created by paclitaxel. Microtubules continuously switch between growth and shortening, a behavior termed dynamic instability. This behavior is required not only for mitotic spindle assembly but also for intracellular trafficking, cell polarity and directed migration. Paclitaxel binds preferentially to polymerized microtubules and, at clinically relevant concentrations, suppresses their dynamics more than simply increases polymer mass. The consequence is a spindle that may appear morphologically present but is unable to satisfy the spindle-assembly checkpoint or generate accurate chromosome attachments (1, 6).

The relationship between mitotic arrest and cell death is not linear. A tumor cell can remain arrested until apoptotic signaling reaches a lethal threshold, die after exiting mitosis, or escape through slippage. The duration of arrest, abundance of pro- and anti-apoptotic proteins, p53 status, cyclin B degradation and basal metabolic state all influence outcome. Paclitaxel can therefore select for cells that tolerate aneuploidy, repair stress more efficiently or activate survival pathways without necessarily altering the drug target itself (2, 5, 28).

### 2.1 Drug transport and intracellular exposure

ABCB1/P-glycoprotein is the best-established transporter associated with taxane resistance. Its overexpression lowers intracellular paclitaxel accumulation and often produces cross-resistance to structurally unrelated substrates. Importantly, the resistant phenotype may involve amplification of a broader chromosome 7q21 region and coordinated expression of multiple genes rather than ABCB1 alone. Other transporters, including ABCC family members and ABCG2 in selected contexts, can contribute, although their importance varies by tumor and formulation (5, 29). Clinical translation of first-generation P-glycoprotein inhibitors was limited by toxicity, pharmacokinetic interactions and inadequate selectivity. Modern approaches are more likely to rely on biomarker-guided combinations, drug formulations less dependent on transporter exposure, or co-delivery systems rather than nonselective systemic pump blockade (3, 4, 29).

### 2.2 Tubulin isotypes and the microtubule interactome

Changes in β-tubulin isotype expression can modify microtubule dynamics and taxane sensitivity. TUBB3 has received particular attention. Experimental upregulation of class III β-tubulin can confer resistance to paclitaxel and vinorelbine, whereas silencing may increase sensitivity. TUBB3 can also counteract the ability of paclitaxel to suppress migration-associated microtubule behavior. Clinical associations have been described in carcinomas of unknown primary site, lung cancer and other tumors, but the predictive value is not universal and can be confounded by tumor lineage and prognosis independent of treatment (30–33). In colorectal models, TUBB3 silencing sensitized HCT116 cells to paclitaxel, supporting the relevance of microtubule composition in a disease where paclitaxel is not routinely used but may be explored in selected experimental or combination settings (33).

Microtubule-associated proteins, plus-end tracking proteins, motor proteins and post-translational modifications further influence taxane response. The resistant microtubule network should therefore be viewed as an ecosystem. A change in one destabilizer may be buffered by changes in tubulin isotypes, acetylation, detyrosination, kinesins or checkpoint proteins. This redundancy is highly relevant to stathmin biology: suppression of STMN1 may restore taxane sensitivity in one model but have a limited effect in another if parallel microtubule-destabilizing programs remain active (1, 2, 6, 7).

### 2.3 Cell-death thresholds and adaptive survival

Paclitaxel-induced death involves mitochondrial apoptosis, death-receptor signaling, mitotic catastrophe and, in some settings, immunogenic consequences. Resistant cells may increase BCL-2 or BCL-XL activity, reduce pro-apoptotic priming, alter caspase activation or engage PI3K–AKT and MAPK signaling. These pathways do not simply sit downstream of microtubule injury. They can also regulate stathmin phosphorylation and stability, thereby changing the physical microtubule response to treatment. This bidirectional relationship helps explain why signaling inhibitors can modify taxane sensitivity even when they do not directly bind tubulin (2, 5, 28, 34).

### 2.4 Kinetochore adaptation, aneuploidy and mitotic slippage

Paclitaxel resistance should not be interpreted as resulting simply from increased aneuploidy. By suppressing microtubule dynamics, paclitaxel can disturb kinetochore-microtubule tension, prolong spindle-assembly checkpoint activation and promote mitotic slippage in cells that fail to execute apoptosis. Chromosome missegregation, micronucleus formation and aneuploid progeny may then provide a substrate for selection during repeated treatment. In this sense, aneuploidy is best viewed as both a potential consequence of paclitaxel-induced mitotic stress and a permissive state that may support resistant evolution, rather than as a universal upstream cause of resistance (35–37).

The kinetochore complex is therefore an important but underexplored layer of paclitaxel resistance. Paclitaxel-treated cells experience abnormal attachment dynamics and altered tension at kinetochore-microtubule interfaces, which are monitored by the spindle-assembly checkpoint. Resistant cell lines may differ in the duration or intensity of checkpoint signaling rather than carrying a single uniform kinetochore defect. Candidate nodes include Aurora B/chromosomal passenger complex-mediated error correction, MPS1-KNL1 signaling, MAD1/MAD2 recruitment, BUB1/BUBR1/BUB3 activity, CDC20/APC/C regulation and NDC80-mediated kinetochore-microtubule attachment. Future studies should therefore measure kinetochore localization of checkpoint proteins, chromosome alignment defects, micronuclei, aneuploidy, cyclin B degradation, mitotic duration and slippage after paclitaxel exposure in sensitive and resistant models (1, 2, 5, 28, 36, 37).

### 2.5 Autophagy and metabolic tolerance

Paclitaxel frequently induces autophagic responses. Autophagy may remove damaged mitochondria, provide metabolites during prolonged stress and delay apoptotic commitment. In many experimental systems, pharmacological or genetic autophagy inhibition enhances paclitaxel cytotoxicity, but autophagy can also contribute to cell death depending on tumor type, dose and temporal context. Consequently, LC3 accumulation alone is insufficient to conclude that protective autophagy is active; flux measurements and functional rescue experiments are required (28, 38, 39). Any proposed link between stathmins and autophagy should therefore be treated cautiously unless flux and causality are directly demonstrated.

### 2.6 Epithelial–mesenchymal plasticity, stemness and the tumor microenvironment

Paclitaxel-resistant populations frequently acquire motile, mesenchymal or stem-like features. These states can combine reduced proliferation with enhanced drug efflux, survival signaling and tumor-initiating capacity. Hypoxia is especially relevant because it can induce TUBB3 through HIF-1α and simultaneously remodel metabolism, apoptosis and immune interactions (40). Cancer-associated fibroblasts, endothelial cells, macrophages and extracellular vesicles may reduce drug delivery or transmit resistance-associated RNAs and proteins. Single-cell analyses increasingly show that resistance is distributed among interacting cell states rather than confined to a stable clone (41–45). The stathmin family intersects this network through cytoskeletal remodeling, cell migration and potentially immune-microenvironmental associations, but the strength of evidence differs markedly among family members.

### 2.7 Formulation, schedule and tumor exposure

The term paclitaxel resistance can obscure major pharmacological differences among solvent-based paclitaxel, albumin-bound paclitaxel and other delivery systems. Formulations alter infusion reactions, systemic exposure, stromal penetration and the contribution of solvent-related toxicity, but they do not abolish the requirement for a vulnerable microtubule network. Schedule also matters. Short high-concentration pulses and prolonged low-concentration exposure can produce different degrees of polymerization, checkpoint activation and cell death. Preclinical resistance studies should therefore report free-drug concentration, exposure duration, vehicle and washout conditions. Without these details, results from different models cannot be compared reliably. In clinical biomarker studies, the exact taxane, dose intensity, combination partner and prior therapy must be incorporated into the analysis because a marker may predict response to one regimen but not another.

### 2.8 Non-coding RNAs and epigenetic adaptation

MicroRNAs, long non-coding RNAs and circular RNAs regulate many components of the resistance network, including ABC transporters, tubulin isotypes, apoptosis, autophagy and epithelial–mesenchymal plasticity. Recent reviews have catalogued numerous paclitaxel-associated non-coding RNAs, but the literature is vulnerable to small sample sizes, inconsistent normalization and limited external validation (42, 43). From the stathmin perspective, non-coding RNAs may influence STMN transcription or the kinases and phosphatases that determine activity. This area is attractive because RNA signatures could serve as minimally invasive biomarkers; however, mechanistic studies must establish direct targets and demonstrate that manipulating the RNA changes paclitaxel response through the proposed stathmin-dependent pathway rather than through a broad stress response.

### 2.9 Resistance as an evolutionary process

Acquired resistance develops under repeated cycles of drug exposure, recovery and repopulation and can involve selection of pre-existing resistant clones, reversible drug-tolerant states, mitotic-slippage-derived aneuploid progeny and stable genetic or epigenetic adaptation (2, 5, 35–37, 41, 45). The surviving population may contain cells with high transporter expression, altered microtubule composition, increased tolerance of checkpoint stress or impaired apoptotic commitment. These mechanisms are not mutually exclusive. A stathmin-high state could be constitutive in one clone, transiently induced by treatment in another, or selected indirectly because it supports migration and survival after mitotic stress. Longitudinal sampling is therefore more informative than comparing unrelated sensitive and resistant cell lines. Barcoding, lineage tracing and serial single-cell profiling can reveal whether STMN alterations precede resistance, arise during adaptation or simply mark the final resistant population.

## 3 Stathmin-family biology: shared mechanisms and member-specific features

**Figure 2:**
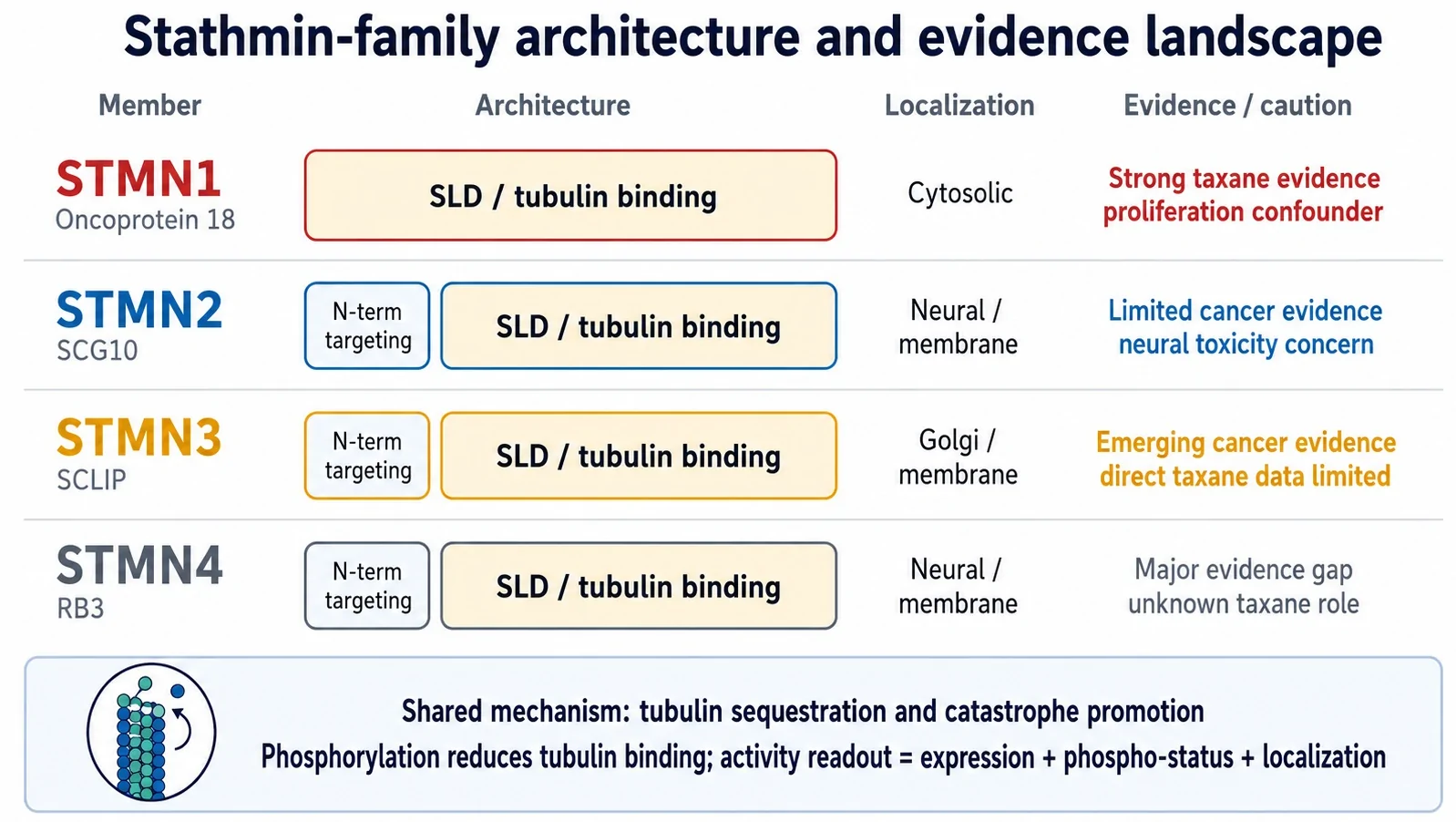
Revised architecture and evidence landscape of the stathmin family. STMN1 contains a stathmin-like tubulin-binding domain and is broadly cytosolic, whereas STMN2, STMN3 and STMN4 contain N-terminal targeting regions that support membrane or Golgi-associated localization. The figure separates structural features, localization, evidence strength, taxane-response evidence and major translational cautions. Phosphorylation reduces tubulin binding, so activity-aware assessment requires total protein, phospho-status and subcellular localization rather than expression alone. The schematic is not drawn to scale.

The four vertebrate stathmin-family proteins are related but not interchangeable. All contain a conserved stathmin-like domain that can interact with two tubulin heterodimers to form a T2S complex. Tubulin sequestration reduces the pool available for polymerization, while additional effects on catastrophe can accelerate microtubule disassembly. Phosphorylation weakens tubulin binding and is therefore a central switch controlling activity. STMN1 contains four well-characterized regulatory serines, whereas STMN2–4 have homologous sites within a longer architecture. STMN2, STMN3 and STMN4 also possess N-terminal membrane-targeting sequences that are subject to palmitoylation and promote localization to the Golgi apparatus, vesicular membranes, axons and growth cones (7–14). The shared and member-specific features of the STMN family are summarized in **Figure 2**. The comparative features of the STMN-family members are summarized in **Table 1**.

**Table 1:** Comparative features of the stathmin family.

Member	Common alias	Localization/structural feature	Cancer evidence	Taxane-response evidence	Major gap
STMN1	Oncoprotein 18	Broadly cytosolic; no long N-terminal membrane-targeting extension	Extensive, multi-tumor	Direct genetic and clinical evidence	Prospective predictive validation; activity-aware assay
STMN2	SCG10	Neural enriched; N-terminal membrane targeting	Limited and lineage dependent	Insufficient	Family-wide functional comparison; neuropathy implications
STMN3	SCLIP	N-terminal membrane targeting; Golgi/neurite localization; STAT3 interaction	Emerging in glioma, lung, breast, leukemia and colorectal contexts	Direct ovarian-cell mechanism; otherwise limited	Replication, *in vivo* validation, phospho-site and biomarker studies
STMN4	RB3	Neural enriched; membrane-associated family member	Very limited	Insufficient	Basic cancer biology and taxane-response studies

### 3.1 STMN1

STMN1 is widely expressed and strongly linked to proliferative states. It participates in spindle assembly, chromosome segregation, migration and cell-cycle progression. High expression is common in aggressive cancers and often correlates with poor outcome (46). Because STMN1 is soluble and broadly distributed, its overexpression can globally increase microtubule turnover. In cancer cells, the functional consequence depends on phosphorylation state as well as total abundance. Measuring total STMN1 without phospho-site information may therefore miss the fraction that is actively destabilizing microtubules (7–9, 47).

### 3.2 STMN2

STMN2/SCG10 is enriched in the nervous system and has a prominent role in axonal growth and regeneration. Its N-terminal targeting domain localizes it to membranes and growth cones, creating a spatially restricted influence on microtubules. Cancer studies are fewer than for STMN1. Any role in taxane response may be most relevant in tumors with neural differentiation or ectopic STMN2 expression, but direct evidence remains limited. The potential for neurotoxicity is also important: interventions that broadly inhibit neuronal stathmins could aggravate rather than reduce taxane-associated peripheral neuropathy (10, 11).

### 3.3 STMN3

STMN3/SCLIP is expressed beyond the nervous system and regulates axonal branching and dendritic development. It interacts with STAT3, and the interaction can influence STMN3 stability and epithelial morphology. STMN3 has been implicated in glioblastoma growth and motility, non-small cell lung cancer invasion, chronic myeloid leukemia cell survival and rectal tumorigenesis (12–14, 25, 26, 48–53). These findings establish biological plausibility but do not demonstrate a universal oncogenic role. In MCF-7 cells, for example, the STAT3–STMN3 complex helped maintain epithelial morphology, and loss of the complex could promote mesenchymal features, illustrating that the phenotypic direction of STMN3 activity may depend on context (34, 50).

### 3.4 STMN4

STMN4/RB3 is the least characterized in oncology. It is mainly associated with neural tissues and membrane-localized microtubule control. The scarcity of cancer and pharmacology studies should not be interpreted as evidence of irrelevance; it instead identifies a major evidence gap. Studies comparing all four family members under identical conditions are needed to determine whether apparent STMN1 specificity reflects biology or historical research bias.

### 3.5 Regulation by kinase and phosphatase networks

Stathmin activity is shaped by CDKs, MAPKs, PKA, Ca2+/calmodulin-dependent kinases and phosphatases. These enzymes connect extracellular growth signals and cell-cycle checkpoints to microtubule behavior. STAT3 can antagonize stathmin-mediated depolymerization and stabilize STMN3 protein, whereas PPP1R14B stabilizes STMN1 in triple-negative breast cancer. These examples reveal two distinct regulatory layers: acute control of tubulin binding through phosphorylation and longer-term control of protein abundance through stabilization or degradation (21, 34, 50). A clinically useful biomarker may therefore need to combine total protein, phospho-status and upstream regulatory activity rather than rely on a single immunohistochemical score.

### 3.6 Quantifying stathmin activity

Total RNA and total protein are convenient but incomplete measures. A functional assessment should consider inhibitory phosphorylation, subcellular distribution, binding partners and the abundance of soluble versus polymerized tubulin. Fractionation assays can quantify microtubule polymer mass, while live-cell reporters and high-resolution imaging can measure growth and catastrophe rates. Proximity ligation, co-immunoprecipitation and phosphoproteomics can identify context-specific partners. These measurements are particularly important for STMN3 because its membrane targeting and STAT3 interaction may create local microtubule effects that are not reflected by bulk expression. A tumor with moderate total STMN3 but a high active membrane-associated fraction may be more relevant to paclitaxel response than a tumor with high but phosphorylated, inactive protein.

### 3.7 Functional redundancy and compensation

The shared stathmin-like domain raises the possibility that inhibition of one family member triggers compensation by another. Most published studies quantify only the targeted protein, so compensatory changes remain poorly characterized. This issue is especially relevant when interpreting STMN1 knockdown. A strong response may indicate that STMN1 is dominant in that cell type, whereas a weak response may reflect replacement by STMN2, STMN3 or STMN4 rather than absence of a stathmin-dependent mechanism. Family-wide transcript, protein and phospho-protein profiling should accompany perturbation experiments. Double and triple knockouts may be required to reveal hidden redundancy, but these experiments must be balanced against effects on cell fitness unrelated to drug response.

### 3.8 Relationship to proliferation

STMN1 often correlates with Ki-67 and other proliferation markers. Rapidly proliferating tumors may be more dependent on microtubule dynamics, more sensitive to taxanes and simultaneously more likely to express STMN1. This creates a potential paradox: the same marker may indicate aggressive biology, taxane vulnerability and resistance, depending on the comparison group and treatment context. Predictive studies must therefore test a formal biomarker-by-treatment interaction and adjust for proliferation. Merely showing that STMN1-high tumors have worse survival after taxane therapy does not prove that STMN1 caused taxane resistance.

## 4 Evidence linking stathmins to paclitaxel resistance

### 4.1 Direct evidence for STMN1

The most persuasive evidence comes from perturbation experiments. In breast carcinoma cells, stathmin overexpression produced resistance to paclitaxel and vinblastine, while RNA interference reversed resistance and increased drug binding to the microtubule compartment (15). Subsequent models confirmed that high STMN1 can oppose taxane-induced stabilization and that silencing can increase sensitivity in epithelial cancers and retinoblastoma (19, 22–24). These studies support causality, but several qualifications are necessary. First, the magnitude of resistance depends on the baseline microtubule state and the level of STMN1 overexpression. Second, STMN1 affects both paclitaxel-stabilized and vinca-destabilized microtubule drugs, sometimes in opposite ways. Third, strong artificial overexpression may produce effects larger than those produced by endogenous clinical variation.

The mechanistic model should not be described as simple competition for paclitaxel binding. Paclitaxel binds inside assembled microtubules, whereas stathmins bind soluble tubulin and regulate catastrophe. STMN1 may reduce the abundance or persistence of the microtubule state on which paclitaxel acts, alter the dynamic response to drug exposure, or change downstream mitotic signaling. The concept is functional antagonism rather than occupation of the same molecular site (1, 7, 15).

### 4.2 Clinical and tumor-specific evidence

In gastric cancer, high STMN1 was associated with chemoresistance and poor prognosis, and functional experiments supported enhanced paclitaxel sensitivity after STMN1 suppression (16). In non-small cell lung cancer, high stathmin expression, sometimes considered together with TUBB3, was associated with resistance to paclitaxel-containing treatment (17). Endometrial cancer studies suggested that STMN1 protein levels may predict taxane response, although validation in prospective cohorts is needed (18). These results are encouraging but heterogeneous. Assays differ in antibodies, scoring systems, specimen timing and treatment combinations; many studies are retrospective; and STMN1 is also a proliferation marker, which can confound its predictive value with general prognostic aggressiveness.

More recent work has strengthened the mechanistic bridge between upstream signaling and STMN1-dependent taxane resistance. In triple-negative breast cancer, PPP1R14B stabilized STMN1 and promoted progression and paclitaxel resistance. The study is notable because it moves beyond simple expression correlation and identifies a potentially druggable regulatory dependency (21). However, targeting an upstream stabilizer is unlikely to be universally effective, because different tumors may regulate STMN1 through distinct kinases, phosphatases, ubiquitin pathways or transcriptional programs.

### 4.3 STMN3 and paclitaxel: limited but important evidence

The direct evidence for STMN3 is narrower but no longer absent. In ovarian cancer cells, bisphosphorylated PEA-15 sensitized cells to paclitaxel by impairing the microtubule-destabilizing effect of SCLIP/STMN3 (25). This experiment is important for three reasons. It demonstrates that STMN3 can influence the paclitaxel-responsive microtubule state; it identifies post-translational regulation rather than abundance alone; and it suggests that the effect of STMN3 may be manipulated indirectly through interacting proteins. Nevertheless, the study does not establish STMN3 as a general biomarker across cancers, and independent replication, genetic rescue and *in vivo* validation remain necessary.

STMN3 has also been linked to tumor-cell motility and signaling. Coordinated expression of stathmin-family members increased motility in non-small cell lung cancer, while nicotine-driven ID1 signaling induced STMN3 and GSPT1 and promoted invasion (48, 49). STMN3 interacts with STAT3, and experimental studies in breast cancer, glioblastoma and leukemia indicate context-dependent effects on morphology, migration, proliferation and survival (34, 50–52). Rectal-cancer transcriptomic work and a recent narrative review further support broad cancer relevance (26, 53). These observations justify testing STMN3 in paclitaxel resistance, but they should not be presented as proof that AKT, ERK, EMT or cancer stemness are direct downstream pathways of STMN3 in every tumor.

A rigorous model separates three evidence levels. Established: STMN3 binds tubulin, destabilizes microtubules and can modulate paclitaxel sensitivity in an ovarian cancer model. Supported but context dependent: STMN3 interacts with STAT3 and influences morphology or motility in selected cancer cells. Hypothetical: STMN3 broadly drives paclitaxel resistance through AKT/ERK activation, EMT, stemness or immune remodeling. The hypothetical level is testable, but it requires genetic loss- and gain-of-function studies, rescue with tubulin-binding-deficient mutants, phospho-site mapping and drug-response experiments in multiple models.

### 4.4 STMN2 and STMN4

Evidence connecting STMN2 or STMN4 to taxane response is currently insufficient for firm conclusions. Their membrane localization, neural enrichment and shared tubulin-binding domain make a role plausible, but expression alone cannot establish function. Family-wide CRISPR screens, quantitative proteomics and isoform-specific rescue experiments are needed. Such studies should also assess neuronal toxicity, because taxanes commonly cause peripheral neuropathy and neuronal stathmins contribute to axonal maintenance.

### 4.5 A microtubule-centered resistance network

Stathmin activity is likely to cooperate with other mechanisms rather than act alone. ABCB1 determines the amount of paclitaxel reaching the cytosol; TUBB3 and microtubule post-translational modifications shape the drug-responsive polymer; stathmins regulate tubulin availability and catastrophe; checkpoint and apoptotic proteins determine whether altered spindle dynamics become lethal. Hypoxia can simultaneously increase TUBB3, change metabolism and alter immune/stromal signaling (40). Thus, the same STMN1 level may predict resistance in a tumor with high ABCB1 and weak apoptotic priming but not in a tumor with adequate intracellular drug exposure and strong mitochondrial commitment. This network model argues for composite biomarkers rather than a single-marker test.

### 4.6 Relevance to colorectal cancer

Colorectal cancer provides a useful experimental setting for testing the family-wide model even though paclitaxel is not a standard backbone therapy. TUBB3 is expressed in a subset of colorectal neoplasms, and its silencing increased paclitaxel sensitivity in HCT116 cells (33). STMN1 has been linked to colorectal-cancer progression and response to other cytotoxic agents, while STMN3 expression has been detected in rectal-cancer transcriptomic analyses (53). These observations justify mechanistic experiments but not immediate clinical extrapolation. A colorectal program should first establish whether paclitaxel reaches meaningful activity in molecularly selected models, whether STMN3 changes microtubule stabilization at clinically achievable concentrations, and whether the effect extends to organoids and xenografts. Such work could identify a niche combination strategy or reveal a general microtubule mechanism relevant to other agents.

### 4.7 Interpreting negative results

Failure of STMN suppression to sensitize a model can be informative. It may indicate that drug efflux is dominant, that apoptosis remains blocked after microtubule stabilization, that another stathmin member compensates, or that the relevant protein is already inactive through phosphorylation. Negative studies should report target suppression, phospho-status, polymerized-tubulin change and intracellular paclitaxel. Without these controls, a negative viability result cannot exclude a stathmin-dependent mechanism. Publishing such studies would reduce selection bias and help define the tumors in which the axis is clinically meaningful.

### 4.8 An evidence hierarchy for the stathmin–taxane field

Evidence should be ranked according to the question being asked. Expression correlations and survival analyses can establish clinical association but not drug specificity. Isogenic perturbation with rescue can establish causality in a defined model, but not generalizability. Pharmacodynamic measurements of microtubule stabilization can connect the protein to the physical drug response. Resistant xenografts and patient-derived models can test tissue context, while prospective clinical interaction analyses are required to establish predictive utility. A convincing translational claim should ideally span several levels. For example, a high STMN1 score should correlate with poor paclitaxel response, genetic suppression should restore microtubule stabilization and killing, and the same signature should predict differential benefit in an independent treated cohort. No STMN3 program has yet completed this chain. Representative evidence linking stathmin-family proteins to taxane response is summarized in **Table 2**.

**Table 2:** Representative evidence linking stathmin-family proteins to taxane response.

Study/model	Family member	Intervention/observation	Main finding	Interpretation
Breast carcinoma cells (Alli et al.)	STMN1	Overexpression and RNA interference	STMN1 promoted resistance; suppression restored sensitivity	Direct causal evidence
Gastric cancer cohorts and models (Bai et al.)	STMN1	Expression analysis and knockdown	High STMN1 associated with chemoresistance and poor outcome	Clinical association plus functional support
NSCLC patients (Sun et al.)	STMN1	Tumor expression during paclitaxel treatment	High expression associated with resistance	Retrospective predictive evidence
Endometrial cancer (Werner et al.)	STMN1	Protein-level biomarker analysis	Potential predictor of taxane response	Requires prospective validation
Triple-negative breast cancer (Liao et al.)	STMN1	PPP1R14B–STMN1 stabilization axis	Promoted progression and paclitaxel resistance	Upstream druggable dependency
Ovarian cancer cells (Xie et al.)	STMN3/SCLIP	Bisphosphorylated PEA-15	Sensitized cells by impairing SCLIP-mediated destabilization	Direct non-STMN1 mechanistic evidence

The distinction between mechanism and marker is equally important. A protein may be mechanistically involved but unsuitable as a biomarker because it is difficult to measure reproducibly or because its activity depends on phosphorylation. Conversely, an expression signature may predict response without being the causal driver. Therapeutic targeting requires causal dependence and a safe intervention; biomarker development requires analytical validity, reproducibility and clinical discrimination. The two goals can overlap but should not be assumed to be identical.

These distinctions should shape future publication standards. Authors should state whether their primary claim concerns association, prediction, causality or therapeutic vulnerability and select experiments accordingly. Clear evidence grading will prevent promising early observations from being overstated and will make genuine advances—particularly for STMN3, STMN2 and STMN4—easier to recognize and reproduce.

## 5 Therapeutic and biomarker implications

### 5.1 Direct suppression of stathmin expression

RNA interference against STMN1 has repeatedly enhanced sensitivity to microtubule-targeting agents in preclinical models (15, 19, 20). The main translational barrier is delivery. Systemic siRNA must reach tumor cells, escape endosomes and avoid immune activation and off-target silencing. Lipid nanoparticles, polymeric carriers, tumor-targeted ligands and local delivery may improve exposure. Co-encapsulation of paclitaxel and an STMN-directed RNA could synchronize target suppression with drug exposure, but formulation stability, dose ratio and normal-tissue toxicity require optimization. Potential strategies for exploiting the stathmin axis are summarized in **Table 3**.

**Table 3:** Potential strategies for exploiting the stathmin axis.

Strategy	Rationale	Key advantage	Principal limitation
siRNA/shRNA or antisense delivery	Lower STMN abundance before paclitaxel	Direct and sequence-specific	Tumor delivery, endosomal escape, normal-tissue effects
Target upstream stabilizers/interactors	Disrupt PPP1R14B–STMN1, STAT3–STMN3 or related dependencies	Potential small-molecule tractability	Context dependence and pleiotropy
Manipulate phosphorylation	Reduce tubulin binding and destabilizing activity	Activity-focused rather than abundance-only	Kinases/phosphatases have many substrates
Composite biomarker	Combine STMN activity with ABCB1, TUBB3 and apoptosis markers	Reflects network biology	Assay complexity and need for prospective validation
Co-delivery nanocarriers	Synchronize paclitaxel and STMN suppression	Improved tumor exposure and fixed ratio	Manufacturing, biodistribution and toxicity

CRISPR-based approaches are valuable experimental tools but are less immediately applicable as systemic cancer therapy. CRISPR interference or base editing could clarify whether specific phosphorylation sites, membrane-targeting motifs or tubulin-binding residues are required for resistance. In translational development, transient gene suppression may be safer than permanent knockout because stathmins have physiological roles in proliferating and neuronal cells.

### 5.2 Targeting upstream regulators or protein interactions

STMN1 and STMN3 are not classical enzymes with obvious small-molecule pockets. Indirect targeting may therefore be more realistic. PPP1R14B–STMN1 stabilization, STAT3–STMN3 interaction and PEA-15–STMN3 regulation provide examples of druggable upstream or partner-dependent mechanisms (21, 25, 34). Yet the direction of intervention must be context specific. Inhibiting STAT3 may destabilize STMN3 in some cells but also alter epithelial morphology, immune signaling and numerous unrelated survival pathways. Combination studies must therefore demonstrate that sensitization is truly mediated by the stathmin axis using rescue experiments.

### 5.3 Phosphorylation-state manipulation

Because phosphorylation reduces stathmin–tubulin binding, promoting inhibitory phosphorylation could, in principle, favor microtubule stabilization. However, the relevant kinases have many substrates, and prolonged activation may cause unwanted proliferative or survival effects. A more selective strategy would target a stathmin-interacting interface or recruit a phosphatase/kinase locally. Structure-guided peptide inhibitors, proteolysis-targeting chimeras and molecular glues are conceptually attractive but remain early-stage possibilities.

### 5.4 Biomarker development

STMN1 immunohistochemistry is feasible in routine pathology, but a predictive assay must distinguish response prediction from general prognosis. Prospective studies should predefine staining thresholds, treatment regimens, endpoints and interaction tests. Ideally, a composite assay would measure STMN1/STMN3 abundance, relevant phospho-sites, TUBB3 and ABCB1, with tumor lineage and proliferative index included in the model. Liquid-biopsy approaches using extracellular vesicles or circulating tumor cells are attractive but require proof that the measured signal reflects the drug-resistant tumor compartment (42–44).

### 5.5 Choice of taxane formulation and combination partner

Nab-paclitaxel, conventional paclitaxel and newer formulations differ in solvent, pharmacokinetics and tissue distribution, but they ultimately rely on taxane–microtubule interactions. A stathmin-associated resistance phenotype may therefore persist across formulations, although improved intratumoral delivery could partially overcome transporter-mediated resistance. Combination partners should be selected based on the dominant resistance architecture: transporter modulation for low intracellular exposure, BH3-mimetic or survival-pathway targeting for weak apoptotic priming, autophagy modulation for demonstrated protective flux, or stathmin-axis suppression for microtubule destabilization. Empirical multi-drug combinations without biomarker selection risk added toxicity without durable benefit.

### 5.6 Designing a prospective biomarker study

A clinically informative study should enroll patients receiving the same paclitaxel-containing regimen and obtain pretreatment tissue with standardized processing. The primary biomarker should be prespecified, including antibody clone, scoring algorithm and cut point. A nested pharmacodynamic cohort could obtain an early on-treatment biopsy to measure microtubule stabilization and stathmin phosphorylation. The statistical plan should test interaction between biomarker status and treatment effect, ideally against a comparator regimen without a taxane. Progression-free survival, objective response, pathological response where applicable, neuropathy and dose intensity should be captured. Archival-only retrospective studies are useful for hypothesis generation but cannot establish clinical utility.

### 5.7 Therapeutic window, resistant-cell selectivity and normal-tissue biology

Stathmins are not tumor-exclusive proteins. STMN1 is expressed in proliferating normal tissues, whereas STMN2–4 contribute to neuronal microtubule organization. Systemic family-wide inhibition could therefore affect bone marrow, intestinal epithelium or peripheral nerves. This is especially important because paclitaxel already produces myelosuppression and peripheral neuropathy. A successful strategy may require tumor-selective delivery, transient inhibition or targeting of a cancer-specific upstream regulator rather than the conserved tubulin-binding domain. Normal organoids, hematopoietic progenitors and sensory-neuron models should be incorporated early in development.

An additional layer of selectivity may come from resistant-cell features that are partly independent of STMN abundance or activity. Paclitaxel-resistant cells can differ from parental cells in intracellular drug exposure, uptake and efflux proteins, apoptotic priming, BCL-2-family dependence, microtubule post-translational modifications, transcription-factor trafficking, surface-marker expression and drug-tolerant state transitions. These features may determine whether STMN inhibition is sufficient to restore paclitaxel response or whether additional interventions are required. For example, an ABCB1-dominant resistant state may require improved drug delivery or transporter-aware combinations before STMN-axis inhibition can become effective, whereas a tumor with adequate intracellular paclitaxel exposure but high active stathmin activity may be more directly sensitized by STMN suppression. Therefore, selective STMN inhibition should be developed together with biomarkers of drug exposure, apoptotic threshold, microtubule modification status, kinetochore/spindle-checkpoint integrity and tumor-cell state rather than as a uniform strategy for all paclitaxel-resistant tumors (2, 5, 28, 29, 35–37).

## 6 Integrated dynamic-balance model: validation framework and clinical application

The central model proposed in this review is a dynamic-balance framework rather than a single-gene resistance model. Paclitaxel pushes the microtubule system toward persistent polymerization and suppressed dynamics, whereas active stathmin-family proteins shift the system toward soluble tubulin, catastrophe and spatial microtubule remodeling. The final phenotype depends on the balance among intracellular drug exposure, tubulin composition, active stathmin abundance, microtubule post-translational modifications, kinetochore/spindle-assembly-checkpoint integrity, mitotic-slippage tolerance and apoptotic priming.

The model also explains why the same STMN alteration may have different consequences in different tumors. If intracellular paclitaxel exposure is low because drug efflux dominates, stathmin suppression alone may not restore pharmacodynamic microtubule stabilization. If checkpoint adaptation or weak apoptotic priming allows survival after mitotic arrest, a restored microtubule response may still produce slippage rather than death. Conversely, a tumor with adequate drug exposure, high active STMN1 or STMN3, an intact apoptotic program and limited compensatory microtubule destabilization may be particularly vulnerable to STMN-axis sensitization.

To validate this model experimentally, future studies should measure several layers in the same system: intracellular paclitaxel accumulation; soluble and polymerized tubulin fractions; total and phosphorylated STMN1-4; subcellular localization of STMN proteins; microtubule post-translational modifications; live-cell microtubule dynamics; kinetochore localization of SAC proteins; mitotic duration and slippage; apoptotic priming; micronucleus formation; aneuploidy and long-term clonogenic survival. Causality should be tested using loss- and gain-of-function approaches, rescue with tubulin-binding-deficient or phosphorylation-site mutants, and resistant models with documented stability after drug withdrawal.

**Figure 3:**
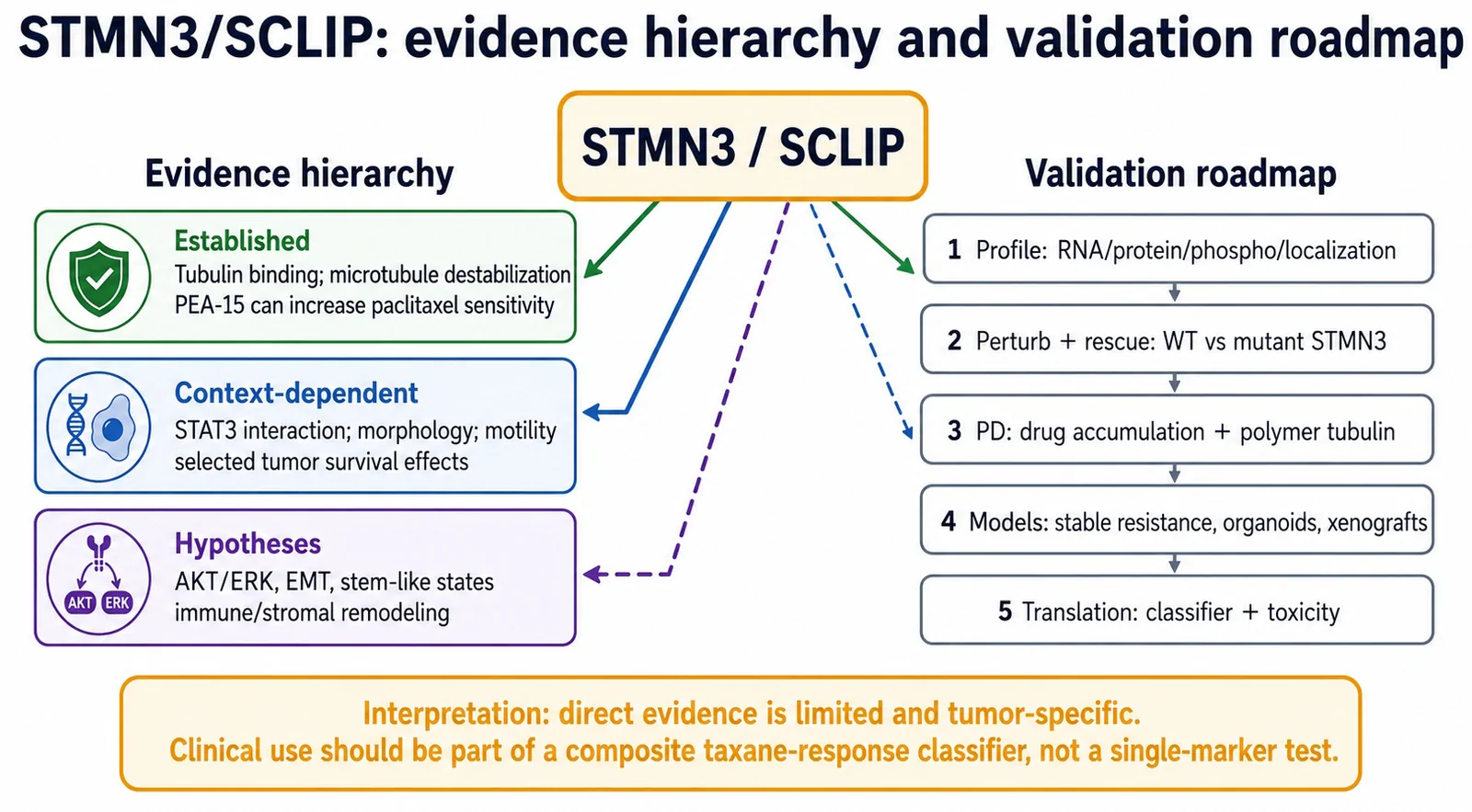
Evidence hierarchy and validation roadmap for STMN3 in paclitaxel response. Established evidence supports tubulin binding, microtubule destabilization and PEA-15-dependent modulation of paclitaxel sensitivity in ovarian cancer cells. Context-dependent evidence links STMN3 to STAT3 interaction, morphology and motility in selected tumor models. Broader links to AKT/ERK signaling, EMT, stem-like states and immune or stromal remodeling remain hypotheses requiring direct validation. The roadmap specifies profiling, perturbation, pharmacodynamic, *in vivo* and biomarker steps needed before clinical translation.

Clinically, the model suggests that stathmin activity should not be used as an isolated biomarker. Instead, it may contribute to a composite paclitaxel-response classifier. Tumors with adequate intracellular paclitaxel exposure, high active STMN1 or STMN3 and preserved apoptotic competence may be most suitable for STMN-axis sensitization. Tumors dominated by drug efflux, weak apoptotic priming, kinetochore/SAC adaptation or protective microenvironmental states may require transporter-aware delivery strategies, apoptosis-sensitizing combinations, mitotic-checkpoint-directed approaches or microenvironment-targeted interventions before STMN inhibition can be effective. This framework converts the model from a descriptive scheme into a testable roadmap for experimental and translational research. The STMN3/SCLIP evidence hierarchy and validation roadmap are summarized in **Figure 3**.

## 7 Controversies, limitations and priorities for future research

Several conceptual problems have slowed translation. First, stathmin abundance is often treated as equivalent to activity, although phosphorylation and localization are crucial. Second, studies frequently use nonphysiological overexpression or a single resistant cell line. Third, paclitaxel resistance is sometimes inferred from short-term viability without measuring intracellular drug concentration, microtubule dynamics, mitotic fate or stable resistance. Fourth, many clinical studies are retrospective and cannot separate predictive from prognostic effects.

The literature also contains apparent contradictions. STMN3 can support motility in glioblastoma and lung cancer, yet the STAT3–STMN3 complex can maintain epithelial morphology in breast cancer cells (25, 34, 48–52). Such results are not necessarily incompatible. Membrane localization, phosphorylation, interacting partners and cell lineage may direct the same microtubule regulator toward different outputs. Future studies should therefore report subcellular localization and phospho-status, not only bulk expression.

A priority experimental design would use paired paclitaxel-sensitive and acquired-resistant models from several tumor types, with STMN1–4 quantified at RNA, total-protein, phospho-protein and localization levels. Each family member should then be knocked out individually and in combinations. Rescue should use wild-type proteins and mutants defective in tubulin binding, phosphorylation or membrane targeting. Live-cell imaging of microtubule dynamics, spindle duration and mitotic fate should be combined with paclitaxel accumulation, apoptosis priming and clonogenic survival. These experiments would determine whether a stathmin phenotype is causal, compensatory or merely correlated.

*In vivo* work should avoid relying only on endpoint tumor volume. Pharmacodynamic sampling should confirm target modulation and microtubule stabilization. Patient-derived organoids and xenografts can preserve heterogeneity, while spatial transcriptomics and multiplex imaging can test whether stathmin-high tumor cells occupy hypoxic, invasive or treatment-protected niches. Single-cell RNA sequencing can identify state transitions after paclitaxel, but RNA data should be integrated with protein phosphorylation and functional assays because stathmin activity is heavily post-translational (21, 25, 45).

The recent publication of a dedicated STMN3 cancer review changes the novelty landscape (26). Future family-focused reviews should not simply repeat a catalog of STMN3 expression across tumors. The stronger contribution is a critical, taxane-specific synthesis that compares all family members, defines evidence levels, identifies contradictions and proposes a standardized validation framework. This distinction is also important for journal selection and peer review.

Clinical translation will require prospective biomarker trials. Patients receiving a defined paclitaxel-containing regimen should be stratified by a prespecified stathmin signature, with interaction testing to determine whether the biomarker predicts differential treatment benefit. A purely prognostic association is insufficient. Parallel analysis of neuropathy is essential because neuronal stathmins may affect axonal vulnerability. Finally, any therapeutic strategy must demonstrate selectivity for malignant cells or a tolerable therapeutic window.

From a precision-oncology perspective, the most plausible near-term use of the stathmin axis is not as a solitary target but as one component of a microtubule-response classifier. Such a classifier could integrate drug exposure, tubulin composition, stathmin activity and apoptotic competence. It may guide the choice between a taxane, an alternative microtubule-binding site, a non-microtubule regimen or a rational sensitizing combination.

A minimum reporting framework would improve reproducibility. Studies should specify cell-line authentication, mycoplasma status, paclitaxel formulation, exposure schedule, resistance index, stability of the phenotype after drug withdrawal, intracellular drug concentration, and whether results derive from short-term viability or long-term clonogenic survival. Stathmin experiments should report the exact isoform, antibody, knockdown efficiency, phosphorylation sites and subcellular localization. Rescue experiments should use constructs resistant to the silencing reagent. Animal studies should report randomization, blinding, pharmacodynamic target engagement and toxicity. These details would allow meaningful comparison across laboratories.

Computational analyses also require stronger validation. Associations in TCGA or other public datasets may reflect tumor purity, cell-cycle activity or neuronal contamination. A prognostic signature containing STMN3 does not prove that STMN3 is the causal component. Independent cohorts, protein-level confirmation and perturbation studies are essential. Spatial methods can determine whether expression resides in malignant cells, nerves, immune cells or stromal compartments. This distinction is particularly important for neural-enriched family members.

## 8 Discussion

The stathmin family sits at an unusually direct interface between oncogenic signaling and the physical target of paclitaxel. This position explains the persistent interest in STMN1 as a resistance determinant and creates a rationale for investigating STMN2–4. Yet biological plausibility should not be confused with clinical validation. STMN1 has causal preclinical evidence and several clinical associations; STMN3 has one especially relevant paclitaxel-sensitization mechanism plus broader tumor-biology evidence; STMN2 and STMN4 remain largely exploratory.

A balanced interpretation also recognizes that microtubule destabilization is not always synonymous with resistance. Excessive destabilization can itself impair proliferation or increase sensitivity to microtubule-destabilizing drugs. The effect depends on the agent’s binding site, concentration and baseline dynamics. Stathmin may potentiate some destabilizers while opposing stabilizers, and the relationship can be nonlinear. This drug-class specificity must be considered when designing combinations or interpreting cross-resistance (15, 19, 20).

**Figure 4:**
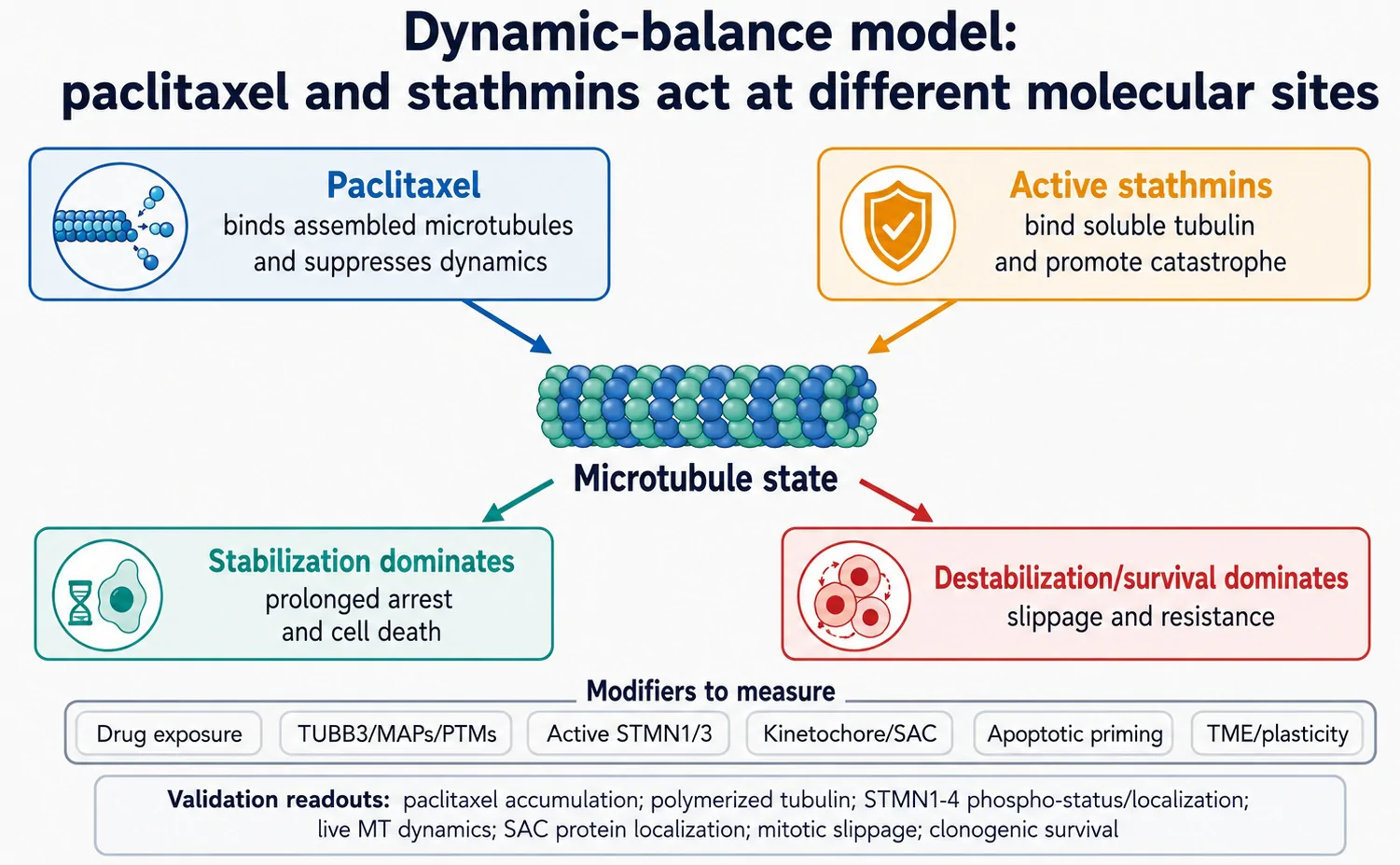
Integrated dynamic-balance model and validation framework. Paclitaxel drives the microtubule system toward a stabilized polymer state, whereas active stathmins shift it toward soluble tubulin, catastrophe and spatial remodeling. The final response is shaped by intracellular drug exposure, tubulin isotypes, microtubule post-translational modifications, active STMN1/STMN3, compensatory STMN family members, kinetochore/SAC integrity, mitotic-slippage tolerance, apoptotic priming and microenvironmental protection. The lower panel summarizes experimental readouts required to validate the model.

The key conceptual advance is therefore a dynamic-balance model. Paclitaxel shifts the microtubule system toward persistent polymer and suppressed dynamics. Active stathmins shift the system toward soluble tubulin, catastrophe and spatial remodeling. This integrated dynamic-balance model is summarized in **Figure 4**. Transporters determine how strongly the drug can push the balance; tubulin isotypes determine the properties of the substrate; survival pathways determine whether the resulting mitotic stress becomes lethal. Resistance emerges when the integrated system remains on the viable side of this balance.

This model generates testable predictions. Tumors with high active STMN1 or STMN3 should show reduced pharmacodynamic microtubule stabilization at equivalent intracellular paclitaxel concentrations. Suppression of the relevant family member should restore stabilization and increase clonogenic killing, and rescue with a tubulin-binding-competent protein should reverse that effect. If sensitization persists with a tubulin-binding-deficient rescue, the mechanism is likely indirect. These experiments can convert a broad association into a mechanistically precise biomarker.

For STMN3, the most defensible conclusion is that it is an emerging regulator rather than a proven pan-cancer driver of taxane resistance. The ovarian PEA-15 study provides direct functional support, while STAT3, ID1 and tumor-expression studies establish context for further investigation (25, 26, 34, 48–53). Claims involving ERK, AKT, EMT, cancer stemness or immune resistance should remain explicitly hypothetical unless directly tested in the relevant model. Such discipline will strengthen, rather than weaken, the novelty of the field by defining the experiments needed to move from inference to evidence.

The family-wide perspective also clarifies why a single therapeutic rule is unlikely to apply across tumors. STMN1-dominant cancers may respond to direct STMN1 suppression, whereas a tumor in which STMN3 is spatially restricted to invasive edges may require a delivery system that reaches that compartment. A tumor with high total STMN1 but strong inhibitory phosphorylation may already have low functional activity and derive little benefit from additional suppression. Conversely, a tumor with moderate expression but high phosphatase activity could have a strongly destabilizing stathmin state. These scenarios argue for a functional assay or a multivariable signature rather than a universal expression threshold.

There is also a need to distinguish sensitization from reversal of stable resistance. An intervention that lowers the paclitaxel IC50 in a parental cell line may be useful but does not demonstrate that it reverses an acquired-resistant phenotype. Stronger evidence includes restoring clonogenic killing in a stable resistant model, preventing tumor regrowth after treatment withdrawal, or re-establishing response in an *in vivo* tumor that has progressed on paclitaxel. Studies should report fold resistance, stability after drug-free culture and cross-resistance to other microtubule agents. This distinction is particularly important for evaluating proposed STMN3 mechanisms, where the current evidence is mainly sensitization rather than clinical reversal of established resistance.

A practical clinical-development pathway can be staged rather than attempting immediate target-directed therapy. The first stage should standardize assays for total and phosphorylated stathmin proteins and determine whether they add information beyond ABCB1 and TUBB3. The second should embed these assays in retrospective cohorts with uniform paclitaxel exposure, using response and treatment interaction rather than overall survival alone. The third should include prospective pharmacodynamic sampling, ideally measuring microtubule polymerization before and after the first treatment cycle. Only after analytical and clinical validity are demonstrated should intervention trials test stathmin-directed sensitization. For STMN3, this sequence is especially important because its neural enrichment creates possible safety concerns and because current tumor evidence is context dependent. Early clinical studies should therefore prioritize biomarker validation and tumor-selective delivery, while monitoring neuropathy, cognition and other neural toxicities. This staged pathway would prevent premature therapeutic claims while allowing a biologically compelling axis to advance through reproducible milestones.

Finally, the microtubule-centered model has implications beyond paclitaxel. Docetaxel, cabazitaxel, epothilones, vinca alkaloids, eribulin and colchicine-site agents perturb microtubules through different binding sites and dynamic effects. A stathmin state that opposes paclitaxel may enhance, reduce or have no effect on another agent. Comparative drug panels can therefore identify whether a resistant tumor should receive an alternative taxane, a destabilizing agent or a non-microtubule therapy. Such experiments may also reveal synthetic vulnerabilities created by stathmin overactivity, converting a resistance mechanism into a treatment opportunity.

## 9 Conclusion

Paclitaxel resistance arises from an integrated network involving drug transport, tubulin composition, microtubule regulators, cell-death thresholds and the tumor microenvironment. Stathmin-family proteins are compelling components of this network because they control the same dynamic microtubule system targeted by paclitaxel. STMN1 has the strongest evidence as a causal resistance factor and candidate biomarker. STMN3 has limited but meaningful direct evidence and should be investigated using rigorous genetic, pharmacodynamic and *in vivo* approaches. STMN2 and STMN4 remain major knowledge gaps. The field should now move from descriptive expression studies toward activity-aware, family-wide and clinically prospective models. Such work may establish stathmin signatures as part of a composite framework for predicting taxane response and selecting rational resistance-reversal strategies.
